# The Role of Virtual Reality in Advancing Surgical Training in Otolaryngology: A Systematic Review

**DOI:** 10.7759/cureus.71222

**Published:** 2024-10-10

**Authors:** Jibril Y Hudise, Mohammed E Mojiri, Ali M Shawish, Khalid A Majrashi, Ahmed Y Ayoub, Atheer M Alshammakhi, Futon A Akoor, Khalid A Madkhali, Maha A Fathi, Rehaf A Areeshi, Ali J Hakami, Ali M Almudawi, Ahmed Saeed M Alqahtani, Mohammed M Akkam, Raghad M Sharahili

**Affiliations:** 1 Otolaryngology, King Faisal Medical City for Southern Region, Abha, SAU; 2 College of Medicine, Jazan University, Jazan, SAU; 3 College of Medicine, King Khalid University, Abha, SAU

**Keywords:** ent procedures, hand-eye coordination, immersive training, medical education, randomized controlled trials, skill acquisition, spatial awareness, surgical performance, surgical training, virtual reality

## Abstract

Technological advancements have rapidly transformed medical education and surgical training, with virtual reality (VR) emerging as a valuable tool. VR offers immersive and interactive environments, enhancing the development of surgical skills without the risks that come with conventional training methods. In this review, we conducted a comprehensive search across PubMed, Web of Science, Scopus, and the Cochrane Central Register of Controlled Trials (CENTRAL), considering all relevant publications up to June 30, 2024. This review focused on randomized controlled trials involving medical students, where VR was used alone or in combination with other educational approaches, compared to traditional training methods. Two independent reviewers extracted data and assessed the quality of studies using the modified Downs and Black scale. Of 1,074 identified articles, six studies met the inclusion criteria. These studies, conducted in Denmark and Australia, utilized a range of VR platforms, including the Visible Ear Simulator, MediseusVR Surgical Drilling Simulator, and Geomagic Haptic device. Quality assessments showed that the studies generally had strong methodology, with reporting scores between 7 and 8 out of 11, and external validity scores between 2 and 3 out of 3. Results indicated that VR training has the potential to improve hand-eye coordination, spatial awareness, and surgical performance in ENT procedures. However, findings on VR’s superiority over traditional methods were mixed, as some studies found no substantial differences in performance metrics. Overall, VR offers a controlled and risk-free training environment that may enhance the acquisition and retention of surgical skills in ENT surgery. Although VR demonstrates significant promise, additional research is needed to fully establish its effectiveness and explore its broader application in surgical education. This systematic review provides a detailed evaluation of VR’s impact on ENT surgical training, highlighting its potential to transform the future of surgical education.

## Introduction and background

The swift advancement of technology has brought about considerable changes in many areas, particularly medical education and surgical training. Among the emerging technologies, virtual reality (VR) stands out as one of the most promising innovations. VR offers the capability to deliver immersive, interactive training experiences that can enhance surgical skills without posing the risks typically associated with traditional training approaches [1].

Otolaryngology, more commonly known as ear, nose, and throat (ENT) surgery, involves procedures that are both delicate and intricate, requiring a high degree of precision and expertise. Historically, surgical training has relied heavily on methods such as cadaver dissections, live surgeries, and theoretical instruction, which have been foundational to medical education for decades. Despite their importance, these methods present certain challenges. For example, the availability of cadaveric specimens is often limited, and ethical concerns can constrain hands-on practice with live patients [2]. The onset of the COVID-19 pandemic has further amplified these challenges by limiting access to clinical environments and resources for education, underscoring the urgent need for alternative training methods [3].

VR presents several key benefits over these traditional training methods. It enables trainees to practice in a safe and controlled environment, where they can repeat procedures, receive instant feedback, and learn from errors without jeopardizing patient safety [4]. VR simulations can encompass a wide variety of surgical scenarios, ranging from routine procedures to more complex or rare cases, thus extending the range of possible training experiences [3,4]. Moreover, VR technology can be tailored to suit an individual’s learning pace, ensuring that trainees attain a high level of competence before managing real patients [3,4].

Numerous studies have shown VR’s effectiveness in improving surgical skills across multiple medical fields, including general surgery, orthopedics, and neurosurgery [1]. In the specific context of ENT surgery, VR has shown promise in enhancing skills such as hand-eye coordination, spatial awareness, and overall surgical performance [5]. Despite these advancements, the application of VR in ENT surgical training has not been thoroughly explored. While existing studies offer some evidence of the benefits of VR in this area [6-15], a systematic review is necessary to gather and analyze these findings comprehensively and to assess the full scope of its effectiveness.

## Review

Methodology

Literature Search Strategy

This systematic review followed the Preferred Reporting Items for Systematic Reviews and Meta-Analyses (PRISMA) guidelines throughout the research process. We conducted a comprehensive search of four major online databases, namely, PubMed, Web of Science (WOS), Scopus, and the Cochrane Central Register of Controlled Trials (CENTRAL), covering publications available up until June 30, 2024. The search terms included combinations of “virtual reality” along with (Otolaryngology OR “Head and Neck Surgery” OR Rhinology OR Laryngology OR Otology OR “Phoniatric Surgery” OR “Sinus Surgery”) using Boolean operators. The search was tailored to each database, with filters applied to include only studies published in English, involving human subjects, and categorized as randomized controlled trials (RCTs). In addition to the database search, we also manually reviewed the reference lists of the selected articles to identify any other potentially relevant studies that were missed in the initial search.

Eligibility Criteria

Selection criteria were developed using the PIOCS framework (P-population, I-intervention, C-comparison, O-outcome, S-study design). We included only randomized clinical trials published in English that (1) involved medical students, (2) implemented VR as a standalone or combined educational tool, (3) compared VR to conventional training methods such as verbal instruction or instructional videos, and (4) used any outcome measure to evaluate the effects of VR. We excluded observational studies, non-English studies, and conference abstracts that lacked full-text versions.

Study Selection

Two reviewers independently screened the titles and abstracts of all identified articles based on predefined eligibility criteria. In cases of disagreement, a third reviewer was consulted to help reach a consensus.

Data Extraction

Full texts of the included studies were then reviewed, and relevant data were extracted. This data included sample size, the specific VR technology used, the age of the participants, intervention details for both experimental and control groups, outcome measures, and key findings. Any disagreements during this stage were resolved by a third reviewer.

Quality Appraisal

The methodological quality of the included studies was assessed by two reviewers independently using the modified Downs and Black scale for clinical trials. This scale evaluates 27 items across the following four domains: reporting, external validity, internal validity, and statistical power. A study was rated as excellent if it scored between 26 and 28 points, good if it scored between 20 and 25, fair if it scored between 15 and 19, and poor if it scored below 15. Any disagreements between reviewers were resolved through discussion until consensus was achieved.

Results

Study Selection

**Figure 1:**
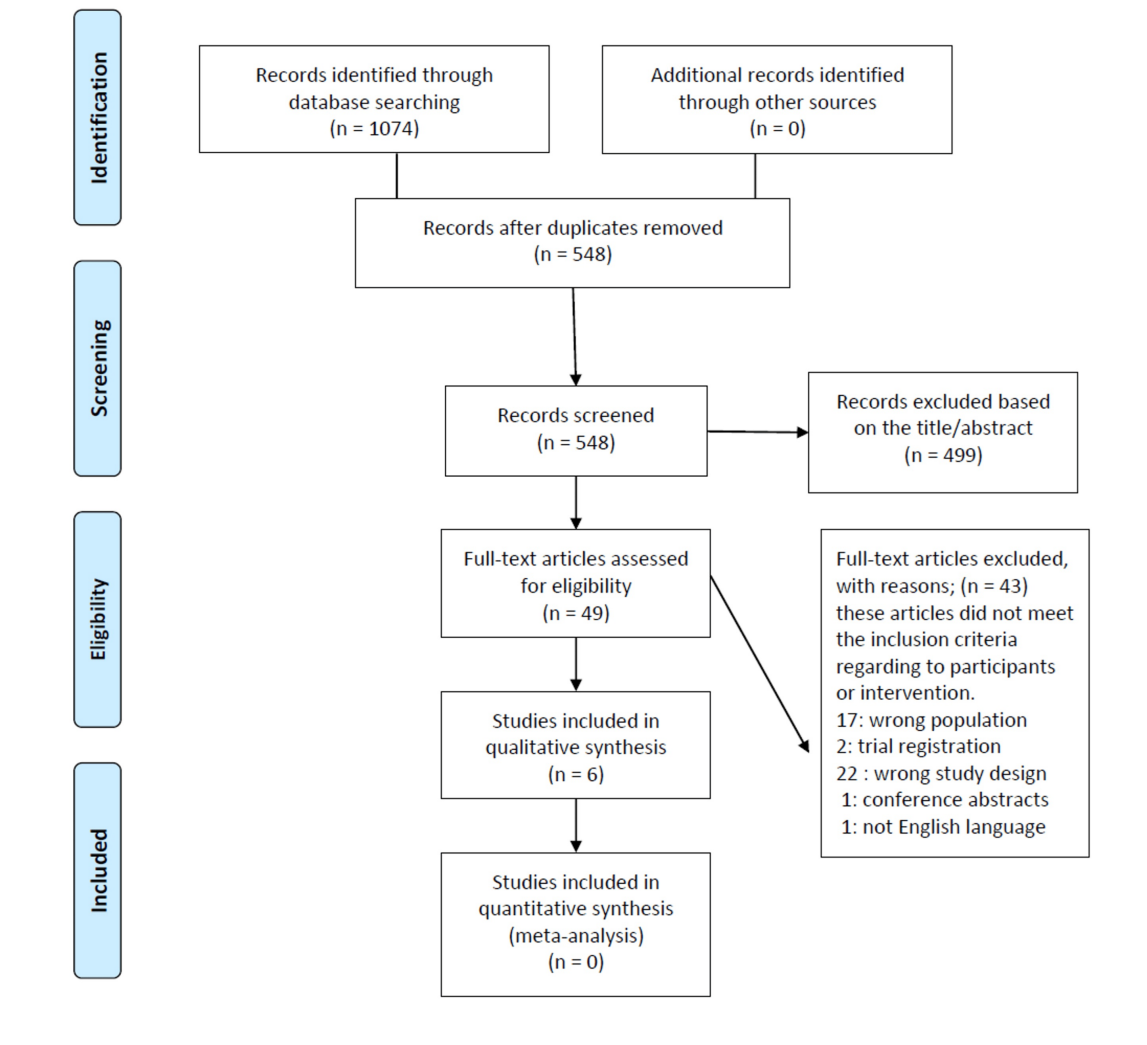
Preferred Reporting Items for Systematic Reviews and Meta-Analyses (PRISMA) flowchart of study search and selection.

For this systematic review, a comprehensive search strategy was implemented using four databases, namely, PubMed, Scopus, Web of Science (WOS), and the Cochrane Library. The search query included the terms “virtual reality” AND (Otolaryngology OR “Head and Neck Surgery” OR Rhinology OR Laryngology OR Otology OR “Phoniatric Surgery” OR “Sinus Surgery”). This thorough approach identified a total of 1,074 papers: 371 from PubMed, 310 from Scopus, 328 from WOS, and 65 from Cochrane. After removing 526 duplicates, 548 papers were left for initial screening. The titles and abstracts of these papers were evaluated, leading to the exclusion of 499 studies. During the full-text review, 43 articles were eliminated for various reasons: 17 studies focused on the wrong population, 22 had inappropriate study designs, two were trial registrations, one was not in English, and one was a conference abstract. Ultimately, six studies satisfied the inclusion criteria and were included in the qualitative synthesis [6-11]. Figure 1 presents the PRISMA flowchart detailing the study selection process.

Study Characteristics

The included studies were conducted across multiple countries, notably in Denmark and Australia, showcasing a global interest in employing VR technology for enhancing surgical skills (see Tables 1, 2). Frendo et al. and Frithiof et al. [7] performed their RCTs in Denmark, focusing on cochlear implant surgery and mastoidectomy, respectively. In the study by Frendo et al., 18 otolaryngology residents, averaging 33.9 years of age, utilized the Visible Ear Simulator (VES) version 3.0. Frithiof et al. included 24 medical students without prior temporal bone simulation training, employing a sophisticated setup that included the VES, Geomagic Haptic device, and ARRISCOPE digital microscope. Andersen et al. also concentrated on mastoidectomy training with 18 novice medical students, utilizing the VES, with the average ages of the control group and experimental group being 22.9 and 25.9 years, respectively.

**Table 1:** Baseline characteristics of included studies. Included studies: Frendo et al. [1], Frithiof et al. [7], Andersen et al. [8], Zhao et al. [9], Wijewickrema et al. [10], Wijewickrema et al. [11]. RCT: randomized controlled trial; VR: virtual reality; CG: control group; E1: experimental group 1; MBBS: Bachelor of Medicine, Bachelor of Surgery; MD: Doctor of Medicine; PhD: Doctor of Philosophy

Author(s), year	Country	Design	Type of surgery	Sample size	Age (years)	VR device
Frendo et al., 2022	Denmark	RCT	Cochlear implant surgery	18 otolaryngology residents	33.9 (2.95)	Visible Ear Simulator (VES) version 3.0
Frithiof et al., 2020	Denmark	RCT	Mastoidectomy	24 medical students, with no prior temporal bone simulation training	23.4 (2.3)	VES, Geomagic Haptic device, ARRISCOPE digital microscope
Andersen et al., 2016	Denmark	RCT	Mastoidectomy	18 novice medical students	CG: 22.9 (21.3–24.4); E1: 25.9 (23.5–28.2)	VES
Zhao et al., 2011	Australia	RCT	Cadaver temporal bone dissection	20 novice trainees with minimal temporal bone experience (8 with minimal, 12 with none)	28.2	MediseusVR Surgical Drilling Simulator (SDS)
Wijewickrema et al., 2015	Australia	RCT	Temporal bone surgery (mastoidectomy)	24 medical students from the University of Melbourne (13 MBBS, 10 MD, and 1 PhD); all participants had prior knowledge of the anatomy of the ear but had no surgical experience.	Not specified	VR temporal bone surgery simulator
Wijewickrema et al., 2018	Australia	RCT	Virtual temporal bone surgery	Trial 1: 30 medical students; Trial 2: 20 medical students	Not specified	VR temporal bone surgery simulator

**Table 2:** Characteristics of the included studies. Included studies: Frendo et al. [1], Frithiof et al. [7], Andersen et al. [8], Zhao et al. [9], Wijewickrema et al. [10], Wijewickrema et al. [11]. VR: virtual reality; CISAT: Computerized Interactive Surgical Assessment Tool; UHF: ultra-high-fidelity; CL: cognitive load; CG: control group; E1: experimental group 1; E2: experimental group 2; P: probability value (used in statistical analysis), χ²: chi-square test statistic

Author(s), year	Intervention E1	E2	Intervention of CG	Dosage	Outcome measures	Results
Frendo et al., 2022	Mastoidectomy including cochlear implantation VR training	-	Mastoidectomy VR simulation training alone	Two hours of additional VR simulation training focused on cochlear implantation, following three hours of VR mastoidectomy training and lectures on temporal bone surgery	• Primary: Surgical performance using CISAT • Secondary: self-directedness (need for assistance)	The intervention group scored 22.9/44 points; the control group scored 21.8/44 points (p = 0.51). The intervention group required assistance 1.3 times during cadaver drilling; the control group required assistance 1.9 times (p = 0.21). No significant improvement in performance or self-directedness
Frithiof et al., 2020	Ultra-high-fidelity (UHF) VR simulation training	-	Conventional screen-based VR simulation training	Each participant performed four mastoidectomy procedures: two under UHF conditions using a digital microscope, and two under conventional conditions using screen-based VR	• Performance (final-product performance score) • CL	UHF VR simulation training resulted in lower performance scores and higher CL compared to conventional VR simulation. Specifically, the mean difference in performance was 1.0 points (p = 0.02); CL was 28% vs. 18% (p < 0.001)
Andersen et al., 2016	CL theory-based instructions with a worked example followed by a problem-completion exercise	-	Standard instructions	One hour of self-directed virtual reality simulation training	• CL (estimated by reaction time testing) • Final-product performance (assessed by blinded expert raters)	Increased CL during post-training procedures (52% vs. 41%, p = 0.02); lower final-product performance score (13.0 vs. 15.4, p < 0.005) for the intervention group
Zhao et al., 2011	VR simulator training	-	Traditional teaching methods (tutorials, videos, models)	Two hours of didactic teaching + two hours of VR simulator training	Performance in cadaveric temporal bone dissection (end product score, injury size, overall performance, technique)	VR group had better end product (80% vs. 45%, p < 0.001), smaller injuries (19% vs. 36%, p = 0.01), better overall performance (55% vs. 35%, p = 0.04), no difference in technique score.
Wijewickrema et al., 2015	Automated feedback during virtual cortical mastoidectomy	-	No automated feedback during virtual cortical mastoidectomy	Participants received automated feedback (or no feedback for the control group) during the simulation session	• Effectiveness of the feedback system in modifying stroke technique • Accuracy of the feedback system • Usability and usefulness of the feedback based on participants’ self-reports	Significant improvement in the drilling performance of the feedback group (Friedman’s test: x²(1) = 14.450, p < 0.001). The feedback system provided timely feedback 88.6% of the time and appropriate feedback 84.2% of the time. Participants’ opinions about the usefulness of the system were highly positive
Wijewickrema et al., 2018	Full visual presentation of drillable areas	Step-by-step visual presentation of drillable areas	-	E1: Full visual presentation, used 3.73% of the time. E2: Step-by-step visual presentation, used 60.40% of the time	• Quality of dissection assessed using the Welling scale	E1: Participants who received a full visual presentation showed a significant improvement in performance (p = 0.03). E2: Participants who received step-by-step visual presentation showed a significant improvement in performance (p < 0.001). Overall finding: The step-by-step presentation was more engaging and used more frequently by participants, indicating it was more likely to be used when given the option

In Australia, Zhao et al. [9] conducted an RCT involving 20 novice trainees with minimal experience in cadaver temporal bone dissection, using the MediseusVR Surgical Drilling Simulator, with an average participant age of 28.2 years. Wijewickrema et al. conducted two studies in 2015 and 2018, further exploring VR in temporal bone surgery [10,11]. The 2015 study included 24 medical students from the University of Melbourne, all possessing prior anatomical knowledge but lacking surgical experience, and utilized a VR temporal bone surgery simulator. The 2018 study comprised two trials: one with 30 medical students and another with 20, employing a VR temporal bone surgery simulator, though specific age details were not disclosed.

The VR technologies utilized across these studies were diverse, reflecting advancements in the field. The VES was primarily employed in Danish studies, underscoring its effectiveness in otolaryngology training. The integration of the Geomagic Haptic device and ARRISCOPE digital microscope in Frithiof et al.’s study enhanced the immersive and tactile experience [7]. The MediseusVR Surgical Drilling Simulator featured in Zhao et al.’s study provided a tailored tool for temporal bone dissection [9]. The studies conducted by Wijewickrema et al. utilized VR temporal bone surgery simulators, demonstrating the increasing dependence on VR technology to create realistic and risk-free surgical training environments [10,11]. Collectively, these studies highlight VR’s potential to enhance surgical education by providing consistent, high-fidelity training opportunities.

Frithiof et al. [7] assessed the effectiveness of ultra-high-fidelity (UHF) VR simulation training in comparison to conventional screen-based VR simulation. Participants executed mastoidectomy procedures under both conditions, revealing that UHF VR training resulted in lower performance scores and increased cognitive load compared to conventional VR training. Zhao et al. [9] illustrated the advantages of combining VR simulator training with traditional educational approaches. Wijewickrema et al. [10,11] emphasized the effectiveness of automated feedback during virtual cortical mastoidectomy training. Their feedback system provided timely and appropriate responses, which participants rated highly in usefulness.

Quality Assessment

The quality assessment of the included studies, evaluated with the modified Downs and Black checklist, displayed variability across different categories, identifying both strengths and areas needing improvement, as depicted in Table 3. Reporting scores ranged from 7 to 8 out of 11, suggesting a generally robust presentation of study hypotheses, outcomes, patient demographics, interventions, confounding factors, findings, variability, probability values, randomization, and blinding techniques. For instance, Andersen et al. and both studies by Wijewickrema et al. achieved high reporting scores of 8, indicating thorough documentation. However, Frendo et al. and Zhao et al. [9] received slightly lower scores of 7, potentially indicating less detailed reporting in specific aspects.

**Table 3:** Quality appraisal scores of the included studies.

Study ID	Total quality appraisal score
Frendo et al., 2022	18
Frithioff et al., 2020	20
Andersen et al., 2016	22
Zhao et al., 2011	18
Wijewickrema et al., 2015	21
Wijewickrema et al., 2018	20

In terms of external validity, most studies scored perfectly, with Frendo et al. [6], Frithiof et al. [7], Andersen et al. [8], and both studies by Wijewickrema et al. [10,11] each achieving a score of 3. This indicates that the subjects and facilities used were representative of a broader population, suggesting the generalizability of the findings. In contrast, Zhao et al. [9] scored slightly lower at 2, reflecting some limitations in the representativeness of their subjects or settings. Nevertheless, the overall high external validity scores imply that the findings from these studies are relevant and applicable in real-world contexts. The evaluation of bias and confounding showed variability among the studies. Andersen et al. [8] attained the highest bias score (6 out of 7), indicating strong efforts in blinding, appropriate statistical methods, and reliable outcome measures. The studies by Wijewickrema et al. [10,11] also demonstrated solid performance with bias scores of 5. In contrast, Frendo et al. [6] and Frithiof et al. [7] scored 4, suggesting potential issues with blinding or other bias-related factors. All studies received a confounding score of 4 out of 6, demonstrating good but not optimal adjustment for confounding variables. Most studies achieved high power scores, with the majority scoring a maximum of 1, except for Frendo et al. [6] and Zhao et al. [9], which received 0, indicating that most studies had adequate power to detect clinically significant effects. These findings collectively suggest that while there are minor areas for enhancement, the included studies generally exhibit strong methodological quality and reliability.

Effect of Virtual Reality on Performance Scores

Frendo et al. [6] reported that the intervention group achieved a mean score of 22.9 out of 44 points, while the control group scored a mean of 21.8 points. This 5.4% difference was not statistically significant (p = 0.51). The 95% confidence interval for the intervention group ranged from 20.5 to 25.4 points, and for the control group, it was 19.3 to 24.2 points. In the drilling components, the intervention group scored a mean of 12.2 points out of a possible 24, compared to 11.5 points for the control group, with this difference also lacking statistical significance (p = 0.63). The 95% confidence interval for the intervention group was 10.1 to 14.3 points, and for the control group, it was 9.4 to 13.6 points. Regarding insertion components, the intervention group recorded an insertion score of 10.7 points, while the control group scored 10.3 points, which again was not statistically significant (p = 0.73). The 95% confidence interval for the intervention group was 9.4 to 12.0 points, and for the control group, it was 9.4 to 11.2 points.

Frithiof et al. [7] revealed that the mean final-product performance score was significantly lower in the UHF VR simulation compared to the conventional VR (cVR) simulation, with a mean difference of 1.0 point out of 17 points (95% confidence interval: 0.2 to 1.7, p = 0.02). Andersen et al. [8] found that the final-product performance was evaluated by two blinded expert raters, revealing that the intervention group had a significantly lower final-product score (13.0) than the control group (15.4), with a statistically significant difference (p < 0.005). Zhao et al. [9] noted that the VR group significantly outperformed the traditional group in dissection end products, achieving an average score of 80% compared to the traditional group’s 45% (p < 0.001). The VR group also outscored the traditional group in overall performance, with averages of 55% compared to 35% (p = 0.04).

Wijewickrema et al. [10,11] indicated that a Friedman’s test revealed significant improvement in drilling performance among the feedback group (χ²(1) = 14.450, p < 0.001). They also reported that the use of procedural guidance led to significant performance enhancements. In a subsequent trial, step-by-step procedural guidance resulted in significant improvements (Kruskal-Wallis, p < 0.001), with participants utilizing this guidance 60.40% of the time.

Effect of Virtual Reality on Cognitive Load

Frithiof et al. [7] found that cognitive load was significantly elevated under the UHF VR condition (28%) compared to the cVR condition (18%), with a statistically significant difference (p < 0.001). Andersen et al. [8] concluded that participants in the intervention group experienced a significantly lower cognitive load during the procedure (p < 0.05), suggesting a beneficial impact of VR training on mental workload.

## Conclusions

This systematic review underscores the significant potential of VR in advancing surgical training within the fields of otolaryngology and head and neck surgery. The integration of VR technology into educational frameworks presents numerous benefits, including the provision of realistic, immersive, and risk-free training environments that can enhance the acquisition of surgical skills. By enabling trainees to practice complex procedures in a controlled setting, VR not only fosters hands-on experience but also aids in developing critical cognitive and technical abilities. Despite these advantages, the effectiveness of VR varies depending on the specific context and application. Differences in study designs, participant demographics, and the types of VR systems employed may contribute to this variability in outcomes. As a result, it is essential to recognize that while VR can be a transformative tool in surgical education, its implementation must be tailored to meet the unique needs of each training environment.
